# Sputum culture conversion definitions and analytic practices for multidrug-resistant TB

**DOI:** 10.5588/ijtld.21.0090

**Published:** 2021-07-01

**Authors:** C. A. Rodriguez, M. B. Brooks, O. Aibana, C. D. Mitnick, M. F. Franke

**Affiliations:** 1 Department of Global Health and Social Medicine, Harvard Medical School, Boston, MA; 2 Department of Internal Medicine, University of Texas Health Science Center – Houston, McGovern Medical School, Houston, TX, USA

Dear Editor,

Conversion of sputum culture from positive for *Mycobacterium tuberculosis* to negative is commonly used to monitor treatment response for rifampin- and multidrug-resistant TB (RR/MDR-TB).^1^,^2^ Sputum culture conversion is also used as a surrogate or proxy for final outcome in observational cohort studies.^3^,^4^ Analyses of culture-based endpoints in observational cohorts requires analytic decisions that can potentially introduce bias and affect study interpretation. For example, only patients with a positive baseline culture are included in conversion analyses, requiring investigators to define “baseline”, i.e., the allowable interval before and/or after treatment initiation a positive culture result is considered for inclusion. Other examples include the operationalization of the conversion definition and the handling of deaths and losses to follow-up (LTFU) occurring prior to conversion. Variability in definitions and analytic practices across studies, and in their reporting, hinders interpretation, assessment of potential bias, and comparisons across studies.

We sought to describe recently published definitions and analytic practices in RR/MDR-TB observational cohorts using culture-based endpoints within three key domains: 1) defining “baseline” cultures, 2) defining and analyzing conversion endpoints, and 3) reporting and analyzing deaths and LTFU occurring prior to conversion. We searched PubMed/MEDLINE for observational cohorts of RR/MDR-TB patients reporting a culture-based endpoint published between January 1^st^, 2015 and December 31^st^, 2020. We focused our search on observational cohorts because, although trials are not immune to bias, factors such as pre-treatment inclusion criteria and enforcement of standardized protocols minimize some opportunity for hypothesized biases. We additionally excluded systematic reviews because they would not include methodologic details of individual studies, studies that did not include a culture conversion endpoint, reviews or case reports, and studies on drug-susceptible TB. We selected a search period spanning 2015–2020 because our objective was to identify recent practices in MDR-TB cohort studies and because the use of culture-based endpoints has increased in recent years. We used search terms related to RR/MDR-TB and culture-based endpoints (e.g., culture-conversion, culture conver^*^) and searched 15 major international clinical and epidemiology journals. A single reviewer abstracted study characteristics, including the objective, whether the study was a retrospective cohort with routinely collected medical records or a prospective cohort with a standard protocol, study setting and size, and the type of culture-based endpoint. Reviewers used a standardized data collection form and met regularly to discuss studies to improve consistency across reviewers. For each domain, we abstracted definitions and analytic practices used and provided relevant quantitative metrics.

Our search strategy yielded 107 publications. We excluded 47 that were results of randomized trials or published study protocols (*n* = 15), systematic reviews or meta-analyses (*n*=12), did not report a culture-based endpoint (*n* = 8), were not on RR/MDR-TB (*n* = 5) or did not have original cohort data (*n* = 7). We reviewed the full text and extracted data from 60 published studies. Seventeen studies (28%) reported on prospective observational cohorts; 43 (72%) were retrospective medical record reviews. Approximately a third of studies reported on <100 patients (Table). Only 4 (7%) studies defined the “baseline” interval for sputum collection before or after treatment initiation. In these, the allowable interval ranged from 6 months pre-treatment to 30 days post-treatment initiation. The proportion without a positive baseline culture (e.g., due to a negative result, contamination, or missingness) was reported in 26 (43%) studies and comprised a median of 18% of the cohort. Forty-five studies (72%) defined the culture-based endpoint used in the study: most commonly (*n* = 21, 35%), this was conversion established by two negative cultures ≥30 days apart; four additional studies required patients could not have a subsequent positive culture. Six studies required only one negative culture to establish conversion. The time-fixed absolute proportion with conversion (e.g., 6 months) and relative survival probability of conversion (i.e., hazard ratio) were the most common outcomes, each reported in 48% of publications.

Only 14 (23%) studies reported how deaths occurring prior to culture conversion were analyzed. In 4 (7%), authors treated deaths as censoring events in time-to-conversion analyses; in 4 (7%), patients who died were excluded; and in 5 (8%) patients who died prior to conversion were considered not to have converted. Approaches were similar for LTFU. Only 2 (2%) studies conducted sensitivity analyses to determine whether a different approach changed results.

**Table:** Published studies’ definitions and analytic practices with culture conversion endpoints in observational cohorts of patients with MDR-TB

	*N*	*n* (%)
Study design and cohort characteristics		
Cohort type	60	
Retrospective clinical or programmatic cohort		43 (72)
Prospective protocolized observational cohort		17 (28)
Cohort size	60	
<25		5 (8)
25–99		14 (23)
100–249		21 (35)
250–499		14 (23)
500–999		3 (5)
≥ 1000		3 (5)
Culture media used	60	
Not reported		30 (50)
Liquid (MGIT, MODS)		8 (13)
Solid (Löwenstein-Jensen)		7 (12)
Liquid (MGIT, MODS) and solid (Löwenstein-Jensen)		15 (25)
Defining “baseline” culture		
Reported number of days *before* treatment that baseline culture can be collected	60	4 (7)
Median (min, max)	4	60 (30, 180)
Reported number of days *after* treatment that baseline culture can be collected	60	5 (8)
Median (min, max)	5	30 (0, 30)
Reported proportion of cohort without baseline culture (due to missingness, contamination or a negative culture result)	60	26 (43)
Median [IQR]	26	18 [9–28]
Reported proportion of cohort with a negative baseline culture	60	12 (20)
Median [IQR]	12	17 [4–31]
Defining and analyzing conversion endpoints		
Culture conversion definition	60	
Not reported		15 (28)
One negative culture^*^		4 (7)
One negative culture without a subsequent positive culture^†^		2 (3)
Two consecutive negative cultures		
Days apart not specified^‡^		6 (10)
14 days apart		2 (3)
15 days apart		1 (2)
26 days apart		1 (2)
28 days apart		2 (3)
30 days apart^§^		21 (35)
Two consecutive negative cultures, without a subsequent positive culture		
Days apart not specified		2 (3)
30 days apart		4 (7)
Endpoint type(s) reported^¶^	60	
Absolute proportions		29 (48)
Relative proportions (e.g., risk ratio, odds ratio)		8 (13)
Absolute survival probability		20 (33)
Relative survival probability (e.g., hazard ratio)		29 (48)
Reported the number of sputum samples collected per study visit according to protocol or standard of care	60	2 (3)
Reported the average number of sputum culture results available per patient over the course of the study	60	2 (3)
Analytic approach to handling contaminated cultures or mycobacterium other than TB	60	
Not reported		55 (92)
Culture results excluded from analysis		4 (7)
Results reported separately as contaminated		1 (2)
Reporting and analyzing death and LTFU occurring prior to conversion		
Death		
Handling of death in primary analysis	60	
Not reported		41 (68)
Censoring event		4 (7)
Excluded		4 (7)
Outcome (i.e., not converted)		5 (8)
Not applicable, no deaths occurred in study period		5 (8)
Competing risk		1 (2)
Reported the proportion of the cohort that died before culture conversion	54	1 (2)
LTFU		
Handling of LTFU in primary analysis		
Not reported		39 (65)
Censoring event		4 (7)
Excluded		7 (12)
Outcome (i.e., not converted)		5 (8)
Not applicable, no LTFU occurred in study period		4 (7)
Competing risk		1 (2)
Reported the proportion of LTFU before culture conversion	56	2 (3)
Sensitivity analyses conducted to account for death or LTFU	60	2 (3)

^*^ *n*=1 study required one negative culture for MDR-TB and pre-XDR-TB patients and two negative cultures 30 days apart for XDR-TB patients; *n*=1 study required one negative culture and clinical or radiological improvement.

^†^ *n* = 1 study included patients with culture-negative *M. tuberculosis* at treatment initiation and considered these patients to have converted if they never had a positive culture during the study period.

^‡^ *n* = 1 study allowed one negative culture with clinical or radiological improvement.

^§^ *n* = 1 study allowed one missing or contaminated culture between negative cultures and considered the inability to produce sputum as a negative culture result.

^¶^ Studies could report >1 type of endpoint.

MDR-TB = multidrug-resistant TB; MGIT = Mycobacteria Growth Indicator Tube; MODS = microscopic observation drug susceptibility; IQR = interquartile range; LTFU = loss to follow-up; XDR-TB = extensively drug-resistant TB.

We identified a stark lack of detail critical to interpreting studies of culture-based endpoints in RR/MDR-TB cohorts. When studies reported these details, there was substantial heterogeneity in definitions and analytic practices. In some contexts, certain definitions and analytic approaches will generate less valid results than others or result in estimates with different interpretations. The use of “baseline” culture intervals extending past treatment initiation can affect study validity by introducing selection bias in cohorts with early deaths and LTFU among patients missing a culture. In fact, a missing or contaminated “baseline” culture was the strongest predictor of early death in a programmatic cohort of drug-resistant TB patients in South Africa.^5^ The decision on how to handle deaths and LTFUs occurring prior to conversion (i.e., exclude them, count them as a non-conversion events, censor them) can alter the interpretation of study findings. When death and LTFU are common, estimates from analyses in which these are treated differently will diverge.

Heterogenous definitions and analytic practices across studies prevents meaningful comparison of findings. Foremost, it is impossible to know whether variation across studies reflect true difference or arise simply from the definitions and analyses imposed. This is particularly important given the popularity of meta-analyses and their influence in determining global guidance for RR/MDR-TB.^6^–^8^ The importance of making valid comparisons across study cohorts also extends to averages ascribed to certain patient populations. For example, the finding that, on average, well under 50% of patients with extensively drug-resistant TB (XDR-TB) have successful outcomes informed the “expected response” for the historical control of the NiX-TB single-arm study.^9^

Several improvements should be made to promote transparency and avoid bias in RR/MDR-TB studies using culture-based endpoints. First, investigators should describe the study inclusion and outcome assumptions that must inevitably be applied to real world data, where patients may not have a pre-treatment culture or monthly follow-up cultures. Second, investigators should conduct thorough sensitivity analyses to ensure study findings are robust to these assumptions and analytic decisions. Similarly, methodologic research, such as simulation studies, can help elucidate the best practices to avoid bias. Finally, as investigators, we must set reporting standards, and as peer reviewers, we must enforce these standards.

The primary limitation of our study is that we did not exhaustively survey the literature. However, our study was not designed to be a comprehensive systematic review. Rather, we purposively sampled international journals that report the majority of published studies on MDR-TB treatment cohorts in order to describe common practices.

While the last decade has provided a wealth of development and trials for new RR/MDR-TB drugs and regimens, clinical questions of drug combinations and duration remain – many of which will not be answered by randomized trials. Evidence from observational cohorts will remain indispensable. Therefore, ensuring the validity of findings from observational studies is of the utmost importance.
